# Supplementary material for: A Likelihood Approach for Real-Time Calibration of Stochastic Compartmental Epidemic Models
Source: PLoS Comput Biol. 2017 Jan 17;13(1):e1005257. doi: 10.1371/journal.pcbi.1005257 (PMC5240920; doi:10.1371/journal.pcbi.1005257)
Supplement: S1 File — (TAR.GZ) [file pcbi.1005257.s014.tar.gz › HSPH_Online-SI-Revision/PF08/n1-severe/n1-severe_PF2_table8.pdf]

1	newly	3 weeks	spec	10.	8.48	{0., 21.}	75.6	0.638648	newly	3 weeks	cumul	63.	29.42	{2., 63.}	58.8254	0.638478	newly	5 weeks	spec	19.	7.16	{0., 18.}	67.7895	0.858472	newly	5 weeks	cumul	93.	43.86	{2., 98.}	60.4731	0.672151	newly	46 weeks	cumul	179.	90.15	{2., 221.}	66.8436	0.784871
2	newly	3 weeks	spec	13.	9.68	{1., 19.}	51.8462	0.445316	newly	3 weeks	cumul	51.	35.44	{7., 61.}	47.1765	0.387605	newly	5 weeks	spec	4.	7.15	{0., 17.}	127.25	0.501234	newly	5 weeks	cumul	64.	51.42	{9., 94.}	49.4375	0.376639	newly	48 weeks	cumul	85.	86.4	{10., 185.}	66.8	0.425355
3	newly	3 weeks	spec	16.	10.49	{1., 21.}	55.5625	0.490222	newly	3 weeks	cumul	56.	35.07	{6., 63.}	50.2321	0.413053	newly	5 weeks	spec	18.	7.14	{0., 17.}	66.2222	0.729562	newly	5 weeks	cumul	91.	51.08	{10., 97.}	53.3187	0.462923	newly	47 weeks	cumul	131.	94.2	{14., 241.}	63.1908	0.497572
4	newly	3 weeks	spec	8.	13.4	{3., 24.}	99.25	0.359707	newly	3 weeks	cumul	27.	47.79	{15., 87.}	96.6296	0.295109	newly	5 weeks	spec	8.	9.44	{0., 20.}	80.25	0.494442	newly	5 weeks	cumul	40.	68.59	{19., 127.}	96.625	0.303432	newly	50 weeks	cumul	56.	122.7	{20., 225.}	148.536	0.388582
5	newly	3 weeks	spec	13.	13.17	{3., 27.}	61.	0.332277	newly	3 weeks	cumul	49.	46.14	{14., 90.}	51.102	0.260414	newly	5 weeks	spec	5.	10.39	{1., 22.}	145.8	0.480195	newly	5 weeks	cumul	61.	68.52	{18., 159.}	59.4098	0.261632	newly	49 weeks	cumul	65.	147.57	{19., 318.}	161.123	0.405174
6	newly	3 weeks	spec	15.	8.46	{0., 17.}	55.2	0.628001	newly	3 weeks	cumul	74.	31.48	{1., 63.}	58.4324	0.679072	newly	5 weeks	spec	23.	6.29	{0., 14.}	72.6522	0.997937	newly	5 weeks	cumul	114.	44.76	{1., 90.}	61.2632	0.735669	newly	46 weeks	cumul	183.	80.26	{1., 188.}	60.5683	0.775677
7	newly	3 weeks	spec	8.	7.12	{0., 17.}	69.5	0.58601	newly	3 weeks	cumul	25.	25.32	{4., 53.}	62.96	0.40341	newly	5 weeks	spec	2.	4.97	{0., 12.}	206.5	0.659223	newly	5 weeks	cumul	27.	36.28	{4., 77.}	85.4815	0.431467	newly	46 weeks	cumul	44.	64.55	{5., 158.}	106.659	0.511312
8	newly	3 weeks	spec	0.1	13.32	{3., 26.}	13222.	1.98318	newly	3 weeks	cumul	0.1	45.93	{14., 86.}	45830.	2.55618	newly	5 weeks	spec	0.1	9.25	{1., 21.}	9166.	1.68558	newly	5 weeks	cumul	0.1	66.15	{18., 127.}	66050.	2.69917	newly	50 weeks	cumul	0.1	118.02	{20., 257.}	117920.	2.88776
9	newly	3 weeks	spec	22.	7.87	{1., 20.}	68.3182	0.76742	newly	3 weeks	cumul	56.	28.01	{4., 62.}	58.1964	0.555094	newly	5 weeks	spec	13.	6.14	{0., 16.}	68.6154	0.819384	newly	5 weeks	cumul	88.	40.94	{5., 91.}	60.9091	0.61374	newly	46 weeks	cumul	139.	73.14	{6., 184.}	65.9856	0.667431
10	newly	3 weeks	spec	10.	7.99	{1., 16.}	56.1	0.416507	newly	3 weeks	cumul	37.	27.4	{5., 50.}	49.0811	0.337463	newly	5 weeks	spec	2.	4.77	{0., 11.}	186.5	0.569219	newly	5 weeks	cumul	45.	38.6	{6., 73.}	52.8889	0.332888	newly	46 weeks	cumul	50.	65.54	{10., 133.}	82.4	0.346672
11	newly	3 weeks	spec	17.	5.65	{0., 14.}	70.1765	0.951818	newly	3 weeks	cumul	63.	19.11	{0., 46.}	70.746	1.0009	newly	5 weeks	spec	10.	3.75	{0., 12.}	75.7	1.1079	newly	5 weeks	cumul	90.	27.01	{0., 68.}	71.6778	1.04468	newly	43 weeks	cumul	187.	57.24	{0., 183.}	73.5615	1.19517
12	newly	3 weeks	spec	0.1	7.31	{0., 16.}	7234.	1.56335	newly	3 weeks	cumul	10.	26.08	{4., 57.}	186.2	0.431544	newly	5 weeks	spec	0.1	5.16	{0., 12.}	5098.	1.31875	newly	5 weeks	cumul	10.	37.35	{5., 78.}	294.1	0.531243	newly	51 weeks	cumul	10.	66.39	{5., 171.}	581.9	0.672025
13	newly	3 weeks	spec	9.	8.98	{1., 19.}	64.4444	0.433335	newly	3 weeks	cumul	45.	32.56	{6., 67.}	48.7111	0.39168	newly	5 weeks	spec	9.	7.57	{0., 17.}	57.4444	0.456782	newly	5 weeks	cumul	69.	47.81	{9., 99.}	48.7971	0.413432	newly	46 weeks	cumul	95.	93.35	{13., 209.}	71.0211	0.475674
14	newly	3 weeks	spec	8.	18.	{5., 32.}	141.75	0.356755	newly	3 weeks	cumul	35.	60.97	{22., 98.}	89.3429	0.260286	newly	5 weeks	spec	7.	12.42	{2., 23.}	112.571	0.376867	newly	5 weeks	cumul	50.	87.83	{31., 144.}	92.14	0.267359	newly	51 weeks	cumul	59.	165.25	{40., 324.}	193.61	0.40406
15	newly	3 weeks	spec	1.	5.76	{0., 16.}	512.	0.743876	newly	3 weeks	cumul	11.	20.34	{1., 45.}	127.455	0.459701	newly	5 weeks	spec	1.	3.93	{0., 11.}	353.	0.703505	newly	5 weeks	cumul	12.	28.81	{1., 66.}	178.917	0.517035	newly	45 weeks	cumul	13.	50.65	{1., 126.}	325.	0.616572
16	newly	3 weeks	spec	17.	8.92	{0., 22.}	64.7059	0.691819	newly	3 weeks	cumul	40.	30.78	{3., 73.}	60.35	0.471174	newly	5 weeks	spec	8.	5.35	{0., 14.}	69.125	0.723276	newly	5 weeks	cumul	60.	42.82	{3., 108.}	61.	0.514071	newly	51 weeks	cumul	89.	78.13	{3., 194.}	76.2809	0.572682
17	newly	3 weeks	spec	3.	7.92	{0., 15.}	197.333	0.54524	newly	3 weeks	cumul	13.	28.44	{4., 57.}	153.077	0.470309	newly	5 weeks	spec	2.	5.16	{0., 12.}	207.	0.630239	newly	5 weeks	cumul	18.	39.62	{4., 80.}	155.444	0.482981	newly	51 weeks	cumul	18.	62.94	{5., 123.}	281.333	0.600193
18	newly	3 weeks	spec	24.	13.86	{4., 27.}	54.5	0.409587	newly	3 weeks	cumul	73.	43.81	{18., 73.}	49.1918	0.328223	newly	5 weeks	spec	26.	11.83	{2., 26.}	61.7308	0.566771	newly	5 weeks	cumul	128.	67.88	{26., 123.}	53.4688	0.383604	newly	51 weeks	cumul	297.	175.87	{36., 403.}	60.8519	0.46494
19	newly	3 weeks	spec	13.	6.15	{0., 14.}	60.2308	0.609555	newly	3 weeks	cumul	52.	21.28	{4., 41.}	60.8462	0.551045	newly	5 weeks	spec	8.	4.21	{0., 11.}	70.625	0.804524	newly	5 weeks	cumul	69.	30.76	{5., 71.}	60.029	0.543629	newly	44 weeks	cumul	105.	55.63	{6., 137.}	66.1619	0.593396
20	newly	3 weeks	spec	5.	6.15	{0., 14.}	93.4	0.565047	newly	3 weeks	cumul	13.	20.89	{1., 48.}	106.692	0.464158	newly	5 weeks	spec	1.	4.32	{0., 12.}	380.	0.689752	newly	5 weeks	cumul	19.	30.33	{1., 67.}	107.105	0.485915	newly	44 weeks	cumul	36.	54.98	{1., 137.}	116.556	0.572211
21	newly	3 weeks	spec	15.	7.84	{0., 18.}	61.4667	0.620028	newly	3 weeks	cumul	44.	27.69	{3., 60.}	54.8864	0.472307	newly	5 weeks	spec	7.	4.85	{0., 12.}	67.2857	0.685592	newly	5 weeks	cumul	62.	38.59	{3., 77.}	55.1774	0.493163	newly	51 weeks	cumul	100.	72.42	{3., 159.}	69.02	0.578637
22	newly	3 weeks	spec	1.	7.54	{1., 16.}	668.	0.77916	newly	3 weeks	cumul	13.	25.69	{3., 52.}	130.846	0.450631	newly	5 weeks	spec	0.1	5.52	{0., 14.}	5452.	1.39995	newly	5 weeks	cumul	13.	37.49	{5., 82.}	214.077	0.539215	newly	45 weeks	cumul	13.	66.98	{6., 154.}	436.462	0.695174
23	newly	3 weeks	spec	12.	10.53	{1., 23.}	62.9167	0.456697	newly	3 weeks	cumul	38.	35.27	{8., 77.}	59.5526	0.34288	newly	5 weeks	spec	6.	7.9	{0., 20.}	93.3333	0.527732	newly	5 weeks	cumul	50.	52.56	{9., 110.}	65.2	0.351344	newly	47 weeks	cumul	82.	111.61	{9., 238.}	98.1098	0.443418
24	newly	3 weeks	spec	7.	7.54	{0., 19.}	88.	0.567692	newly	3 weeks	cumul	21.	24.92	{3., 61.}	82.8571	0.415192	newly	5 weeks	spec	2.	5.48	{0., 16.}	238.	0.688337	newly	5 weeks	cumul	24.	37.14	{4., 88.}	112.	0.433465	newly	44 weeks	cumul	30.	75.73	{5., 189.}	204.033	0.521927
25	newly	3 weeks	spec	3.	4.69	{0., 11.}	113.667	0.511055	newly	3 weeks	cumul	19.	16.64	{1., 33.}	62.2105	0.439382	newly	5 weeks	spec	2.	2.88	{0., 8.}	124.	0.630443	newly	5 weeks	cumul	27.	23.33	{2., 48.}	64.1852	0.463725	newly	42 weeks	cumul	28.	40.33	{3., 97.}	106.679	0.493366
26	newly	3 weeks	spec	6.	5.86	{0., 14.}	78.3333	0.631223	newly	3 weeks	cumul	26.	21.85	{0., 54.}	68.5	0.600073	newly	5 weeks	spec	13.	3.9	{0., 10.}	73.6923	1.01953	newly	5 weeks	cumul	53.	30.08	{0., 72.}	63.5849	0.705499	newly	49 weeks	cumul	100.	67.37	{0., 139.}	76.83	0.806102
27	newly	3 weeks	spec	0.1	11.64	{1., 24.}	11552.	1.79934	newly	3 weeks	cumul	0.1	39.16	{7., 82.}	39060.	2.42969	newly	5 weeks	spec	0.1	8.53	{0., 19.}	8452.	1.58747	newly	5 weeks	cumul	0.1	57.32	{10., 120.}	57220.	2.58368	newly	48 weeks	cumul	0.1	101.27	{14., 203.}	101170.	2.76856
28	newly	3 weeks	spec	18.	16.57	{4., 34.}	56.0556	0.301411	newly	3 weeks	cumul	61.	56.4	{22., 110.}	50.1639	0.248886	newly	5 weeks	spec	6.	14.24	{3., 29.}	164.	0.439857	newly	5 weeks	cumul	78.	86.93	{31., 176.}	56.3205	0.238357	newly	51 weeks	cumul	104.	212.	{40., 469.}	137.173	0.366956
29	newly	3 weeks	spec	6.	5.62	{0., 12.}	73.6667	0.554657	newly	3 weeks	cumul	22.	19.19	{2., 42.}	62.1364	0.471474	newly	5 weeks	spec	6.	4.15	{0., 10.}	73.8333	0.698449	newly	5 weeks	cumul	37.	28.02	{2., 65.}	60.9189	0.524856	newly	44 weeks	cumul	89.	54.35	{2., 147.}	69.0225	0.708486
30	newly	3 weeks	spec	5.	6.42	{0., 16.}	93.6	0.530224	newly	3 weeks	cumul	19.	23.25	{2., 47.}	81.8421	0.459662	newly	5 weeks	spec	1.	4.76	{0., 13.}	430.	0.742454	newly	5 weeks	cumul	23.	33.25	{2., 74.}	97.9565	0.483663	newly	45 weeks	cumul	36.	58.13	{2., 148.}	127.472	0.582448
31	newly	3 weeks	spec	5.	8.57	{0., 20.}	141.8	0.711102	newly	3 weeks	cumul	36.	27.95	{0., 63.}	67.25	0.711965	newly	5 weeks	spec	7.	6.12	{0., 17.}	84.8571	0.788464	newly	5 weeks	cumul	48.	40.84	{0., 98.}	73.5	0.749298	newly	46 weeks	cumul	144.	73.5	{0., 218.}	70.2361	0.975885
32	newly	3 weeks	spec	16.	9.08	{0., 20.}	58.375	0.59464	newly	3 weeks	cumul	65.	32.5	{6., 65.}	55.8154	0.531732	newly	5 weeks	spec	15.	6.77	{0., 16.}	64.6	0.783194	newly	5 weeks	cumul	94.	47.26	{6., 92.}	57.4468	0.561957	newly	48 weeks	cumul	156.	89.82	{6., 221.}	62.2051	0.625249
33	newly	3 weeks	spec	6.	14.	{3., 25.}	152.	0.361916	newly	3 weeks	cumul	27.	45.5	{18., 95.}	85.8519	0.231908	newly	5 weeks	spec	2.	10.36	{1., 20.}	434.	0.647296	newly	5 weeks	cumul	33.	67.52	{23., 142.}	118.182	0.287493	newly	51 weeks	cumul	47.	152.08	{28., 344.}	240.17	0.412778
34	newly	3 weeks	spec	10.	4.99	{0., 12.}	66.9	0.720092	newly	3 weeks	cumul	29.	17.83	{1., 41.}	63.7586	0.584225	newly	5 weeks	spec	9.	3.74	{0., 9.}	69.7778	0.859867	newly	5 weeks	cumul	48.	26.22	{2., 59.}	63.625	0.627079	newly	43 weeks	cumul	102.	50.29	{2., 131.}	69.3627	0.75039
35	newly	3 weeks	spec	0.1	11.98	{3., 23.}	11886.	1.92329	newly	3 weeks	cumul	3.	42.47	{20., 77.}	1315.67	1.08397	newly	5 weeks	spec	0.1	9.03	{1., 20.}	8942.	1.73746	newly	5 weeks	cumul	3.	61.59	{24., 116.}	1953.	1.23712	newly	51 weeks	cumul	3.	122.02	{30., 249.}	3967.33	1.46019
36	newly	3 weeks	spec	0.1	5.17	{0., 12.}	5112.	1.32031	newly	3 weeks	cumul	0.1	19.4	{2., 39.}	19308.	2.03152	newly	5 weeks	spec	0.1	3.53	{0., 8.}	3494.	1.0606	newly	5 weeks	cumul	0.1	27.11	{2., 54.}	27018.	2.14772	newly	52 weeks	cumul	0.1	45.46	{2., 104.}	45368.	2.26978
37	newly	3 weeks	spec	18.	4.33	{0., 12.}	75.9444	1.05028	newly	3 weeks	cumul	45.	15.46	{1., 35.}	67.6	0.760202	newly	5 weeks	spec	10.	3.26	{0., 9.}	73.6	0.991258	newly	5 weeks	cumul	67.	22.06	{2., 55.}	69.1642	0.794631	newly	42 weeks	cumul	100.	42.77	{2., 98.}	71.81	0.813915
38	newly	3 weeks	spec	3.	12.02	{2., 24.}	315.333	0.53319	newly	3 weeks	cumul	8.	41.15	{12., 85.}	414.875	0.593302	newly	5 weeks	spec	0.1	7.99	{1., 17.}	7906.	1.61385	newly	5 weeks	cumul	8.	58.74	{17., 117.}	634.25	0.736318	newly	50 weeks	cumul	8.	97.06	{20., 224.}	1113.25	0.911359
39	newly	3 weeks	spec	6.	7.89	{1., 18.}	84.8333	0.429002	newly	3 weeks	cumul	26.	28.49	{5., 54.}	61.2692	0.351263	newly	5 weeks	spec	6.	5.83	{0., 14.}	81.5	0.630331	newly	5 weeks	cumul	37.	41.2	{6., 84.}	65.9459	0.381208	newly	46 weeks	cumul	74.	72.57	{7., 166.}	70.5811	0.47776
40	newly	3 weeks	spec	9.	8.46	{0., 21.}	73.3333	0.59003	newly	3 weeks	cumul	26.	30.57	{3., 65.}	79.5	0.410183	newly	5 weeks	spec	4.	5.73	{0., 14.}	113.25	0.606939	newly	5 weeks	cumul	37.	43.33	{4., 94.}	78.5135	0.41811	newly	52 weeks	cumul	38.	79.2	{4., 194.}	156.474	0.502104
41	newly	3 weeks	spec	0.1	11.39	{1., 24.}	11300.	1.81473	newly	3 weeks	cumul	9.	38.82	{10., 72.}	336.222	0.537695	newly	5 weeks	spec	0.1	7.98	{0., 21.}	7910.	1.52069	newly	5 weeks	cumul	9.	56.09	{12., 111.}	527.667	0.668739	newly	49 weeks	cumul	9.	91.61	{12., 213.}	922.333	0.824674
42	newly	3 weeks	spec	13.	6.84	{0., 14.}	64.7692	0.705572	newly	3 weeks	cumul	42.	24.65	{1., 54.}	63.881	0.636584	newly	5 weeks	spec	12.	4.67	{0., 11.}	73.4167	0.933923	newly	5 weeks	cumul	62.	34.5	{1., 69.}	63.6774	0.664878	newly	49 weeks	cumul	154.	73.14	{1., 134.}	76.039	0.852579
43	newly	3 weeks	spec	2.	5.21	{0., 15.}	227.5	0.723639	newly	3 weeks	cumul	22.	17.22	{0., 43.}	67.5455	0.679731	newly	5 weeks	spec	4.	3.81	{0., 10.}	90.75	0.755978	newly	5 weeks	cumul	31.	25.38	{0., 63.}	70.4516	0.707917	newly	42 weeks	cumul	85.	48.6	{0., 123.}	70.	0.903412
44	newly	3 weeks	spec	4.	15.87	{4., 32.}	305.75	0.526649	newly	3 weeks	cumul	11.	54.03	{19., 105.}	391.909	0.60363	newly	5 weeks	spec	3.	12.46	{1., 23.}	335.333	0.620865	newly	5 weeks	cumul	24.	80.94	{23., 155.}	243.083	0.460406	newly	50 weeks	cumul	29.	165.04	{33., 379.}	473.448	0.609934
45	newly	3 weeks	spec	11.	13.56	{1., 32.}	78.	0.385659	newly	3 weeks	cumul	31.	43.07	{9., 99.}	77.8387	0.302581	newly	5 weeks	spec	14.	9.97	{0., 23.}	69.0714	0.605064	newly	5 weeks	cumul	50.	64.46	{10., 145.}	74.88	0.318252	newly	47 weeks	cumul	85.	139.66	{12., 299.}	113.035	0.408661
46	newly	3 weeks	spec	18.	7.58	{0., 15.}	63.	0.768323	newly	3 weeks	cumul	58.	27.61	{2., 55.}	54.569	0.573533	newly	5 weeks	spec	11.	5.91	{0., 15.}	63.	0.767329	newly	5 weeks	cumul	91.	40.67	{4., 82.}	56.7802	0.622027	newly	46 weeks	cumul	196.	76.81	{4., 173.}	63.9133	0.773565
47	newly	3 weeks	spec	0.1	12.84	{3., 25.}	12744.	1.96763	newly	3 weeks	cumul	0.1	48.42	{20., 84.}	48320.	2.59568	newly	5 weeks	spec	0.1	9.07	{2., 18.}	8980.	1.77995	newly	5 weeks	cumul	0.1	68.11	{24., 127.}	68010.	2.73626	newly	52 weeks	cumul	0.1	106.72	{29., 198.}	106620.	2.89354
48	newly	3 weeks	spec	5.	10.4	{1., 21.}	147.6	0.495631	newly	3 weeks	cumul	29.	35.86	{5., 72.}	73.8621	0.350422	newly	5 weeks	spec	5.	7.84	{0., 17.}	119.6	0.584755	newly	5 weeks	cumul	37.	52.49	{5., 116.}	88.9459	0.384737	newly	46 weeks	cumul	57.	110.28	{5., 275.}	144.491	0.484143
49	newly	3 weeks	spec	13.	5.95	{0., 14.}	67.9231	0.717522	newly	3 weeks	cumul	31.	22.39	{3., 51.}	59.6452	0.475196	newly	5 weeks	spec	3.	4.08	{0., 8.}	104.667	0.568545	newly	5 weeks	cumul	42.	31.13	{3., 70.}	59.0714	0.465055	newly	50 weeks	cumul	46.	53.9	{4., 124.}	85.6522	0.473602
50	newly	3 weeks	spec	11.	9.02	{0., 21.}	59.8182	0.49441	newly	3 weeks	cumul	42.	32.24	{6., 64.}	50.5714	0.412839	newly	5 weeks	spec	10.	6.87	{0., 16.}	64.5	0.617793	newly	5 weeks	cumul	58.	47.	{6., 96.}	52.2414	0.424349	newly	48 weeks	cumul	98.	91.13	{6., 215.}	71.7449	0.52832
